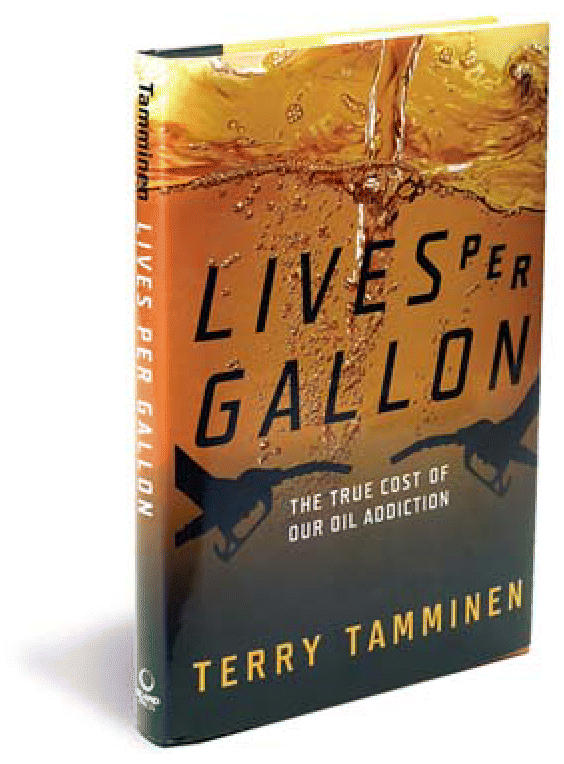# Lives Per Gallon: The True Cost of Our Oil Addiction

**Published:** 2007-02

**Authors:** Peter W. Preuss

**Affiliations:** Peter W. Preuss is the Director of the National Center for Environmental Assessment at the U.S. Environmental Protection Agency (EPA). This internationally known Center prepares many of the hazard and dose–response assessments (known as the Integrated Risk Information System) for EPA Programs, and develops new methods for improving risk assessment. This Center also prepares the National Ambient Air Quality Criteria Documents that are used as the scientific basis for decisions under the Clean Air Act

By Terry Tamminen

Washington, DC:Island Press, 2006. 262 pp. ISBN: 1-59726-101-7, $24.95

*Lives Per Gallon*, by Terry Tamminen, is an easy and entertaining read. It tells an important story about our addiction to oil and the price we are paying to support our habit. It is clear from the very first sentence in the book that the author’s heart is in the right place, and that he has the experience of having been both a senior public advocate and the Secretary of the California Environmental Protection Agency to lend gravitas to his voice.

*Lives Per Gallon* clearly demonstrates how our country’s public and private policies have led us to the situation in which we now find ourselves. Congress, the executive branch of the government, the oil and automobile industries, as well as many others, are examined, scrutinized, and called to account. It is a book that is meant to make our collective hair stand on end—and it succeeds! Tamminen describes the life cycle of oil, from finding it to burning it and disposing of the residues, and describes the hazards and risks associated with the many steps in the process. Finally, he describes the steps that he believes are necessary to get us clean again.

Nevertheless, I must admit that I found portions of the book somewhat distressing to read. Although I clearly understood that I was reading a trumpet call to action rather than an objective scientific presentation, it disturbed me that so many of the endnotes for each chapter were citations to secondary and even tertiary sources (so-and-so scientist, as quoted in such-and-such a newspaper). Looking up some of these citations on the web, I found that they ranged from student papers to newspaper articles, and from presentations by advocacy groups to articles published in the peer-reviewed literature. The latter were unfortunately in the minority, so it proved very difficult to accept the accuracy and validity of some of the “facts” and conclusions. The scientific case in *Lives Per Gallon* could have been better made with the use of more primary sources. Selecting specific citations to make a point is accepted in advocacy, I imagine, but it left me uneasy when it came to discussions of the science. For example, the book would have been better served by a little more attention to toxicology and its principles. The dose does make the poison, and the author often inappropriately equates hazard with risk.

Nevertheless, I think that this is an important book. It describes one of our country’s most pressing problems in detail, and then provides, in equal detail, steps to improving and even solving the problem. Many of the steps that Tamminen recommends have been obvious for a long time, but for many reasons have not been implemented—and deserve to be repeated here. Others deal with newer technology. The major steps Tamminen lays out are conservation, fuel efficiency, and evolution to hydrogen fuel. He discusses this last step in depth, showing us how hydrogen may in fact be the solution, and the steps that we need to take to get there.

This is a book that everyone should read, especially our elected officials from both parties and our captains of industry.

## Figures and Tables

**Figure:**